# Robot-assisted unicompartmental knee arthroplasty can reduce radiologic outliers compared to conventional techniques

**DOI:** 10.1371/journal.pone.0225941

**Published:** 2019-12-03

**Authors:** Kwan Kyu Park, Chang Dong Han, Ick-Hwan Yang, Woo-Suk Lee, Joo Hyung Han, Hyuck Min Kwon

**Affiliations:** Department of Orthopedic Surgery, Yonsei University College of Medicine, Seoul, Korea; Monash University, AUSTRALIA

## Abstract

**Background:**

The aim of this study was to compare the clinical and radiologic outcomes of robot-assisted unicompartmental knee arthroplasty (UKA) to those of conventional UKA in Asian patients.

**Methods:**

Fifty-five patients underwent robot-assisted UKA and 57 patients underwent conventional UKA were assessed in this study. Preoperative and postoperative range of motion (ROM), American Knee Society (AKS) score, Western Ontario McMaster University Osteoarthritis Index scale score (WOMAC), and patellofemoral (PF) score values were compared between the two groups. The mechanical femorotibial angle (mFTA) and Kennedy zone were also measured. Coronal alignments of the femoral and tibial components and posterior slopes of the tibial component were compared. Additionally, polyethylene (PE) liner thicknesses were compared.

**Results:**

There was no significant difference between the two groups regarding postoperative ROM, AKS, WOMAC and PF score. Robot group showed fewer radiologic outliers in terms of mFTA and coronal alignment of tibial and femoral components (*p* = 0.022, 0.037, 0.003). The two groups showed significantly different PE liner thicknesses (8.4 ± 0.8 versus 8.8 ± 0.9, *p* = 0.035). Robot group was the only influencing factor for reducing radiologic outlier (postoperative mFTA) in multivariate model (odds ratio: 2.833, p = 0.037).

**Conclusion:**

In this study, robot-assisted UKA had many advantages over conventional UKA, such as its ability to achieve precise implant insertion and reduce radiologic outliers. Although the clinical outcomes of robot-assisted UKA over a short-term follow-up period were not significantly different compared to those of conventional UKA, longer follow-up period is needed to determine whether the improved radiologic accuracy of the components in robotic-assisted UKA will lead to better clinical outcomes and improved long-term survival.

## Introduction

Medial unicompartmental knee arthroplasty (UKA) is being used more often for surgical treatment of isolated medial compartmental osteoarthritis of the knee [1–3]. UKA has the benefits of preserving both cruciate ligaments and maintaining nearly normal kinetics, in addition to yielding better functional outcomes, preserving more bone stock, enabling more rapid recovery, and causing less blood loss compared to total knee arthroplasty (TKA) [4–7]. However, UKA has not been widely used due to its technically demanding procedures, which are especially difficult for beginners, such as its minimally invasive techniques and limited surgical exposure [7–10]. Moreover, inaccurate prosthesis positioning in UKA has been associated with high failure rates and poor outcomes, leading to concerns about this technique [6,11,12]. Several authors have reported that the surgical outcomes can be improved and the failure rate decreased in UKA by precise prosthesis positioning and fixation [13,14].

Computer-based navigation and robot-assisted UKA have achieved excellent postoperative outcomes and improved prosthesis positioning [15–19]. In particular, robot-assisted UKA with a dynamic referencing tactile-guidance robotic system has been shown to achieve more precise component positioning, because it is based on preoperative 3D computed tomography (CT) [20,21]. Several studies have reported that robot-assisted UKA achieves improved prosthesis positioning and more objective dynamic soft tissue balancing compared to conventional UKA [3,17,18,21]. Additionally, robot-assisted UKA does not have a learning curve regarding the accuracy of implant and clinical outcomes for beginners [10]. Since most of the poor outcomes of UKA are associated with inaccurate prosthesis position and malalignment, robot-assisted UKA is expected to yield better outcomes, and several studies have reported excellent outcomes of robot-assisted UKA in terms of reducing revision rate and limb alignment outliers [22,23]. Compared to conventional UKA, robot-assisted UKA also showed kinematic benefits during gait and excellent clinical score [24–26]. However, only a few studies have compared the clinical and radiologic outcomes of robot-assisted UKA to those of conventional UKA [27]. Knee morphologic features of tibia and femur in Asians are different from those of Caucasians, in terms of size and shape [28]. However, no studies have looked at the surgical outcomes of robot-assisted UKA in Asian patients. Therefore, the purpose of this study was to compare the clinical and radiologic outcomes of robot-assisted UKA to those of conventional UKA in Asian patients. We hypothesized that robot-assisted UKA would provide more accurate prosthesis positioning and alignment, as well as better short-term clinical outcomes.

## Materials and methods

The data collection methods and research design were approved by the Institutional Review Board (IRB) of Severance Hospital (IRB # 2016–0017). Since this study was a retrospective comparative study, informed consent was waived by the ethic committee.

### Patient recruitment

Fifty-five (55) consecutive patients who underwent medial UKA using robotic-assisted bone preparation with a tactile guidance system (Mako, Stryker Corp., Mahwah, NJ, USA) and fifty-seven (57) consecutive patients who underwent medial UKA using conventional surgical procedures between March 2016 and February 2017 were included in the study. From November 2015, the medial UKA using robotic-assisted bone preparation with a tactile guidance system has been considered standard surgical treatment of UKA at our institution. However, robotic-assisted medial UKA costs about $1,800 more than conventional surgery in our institution. Some patients refused the robotic-assisted UKA due to economic problem and wanted to receive the conventional UKA. Therefore, the comparison between patients who underwent robotic-assisted UKA and patients who underwent conventional UKA could be possible.

All patients had medial knee pain clinically attributed to isolated medial compartment arthritis or osteonecrosis. Patients with radiologic evidence of moderate to severe osteoarthritis in the lateral or patellofemoral compartments and those with anterior cruciate ligament deficiency were excluded from this study. Patients with lower extremity fixed deformities such as severe varus or a valgus knee deformity of greater than 10 degrees, previous surgery, secondary osteoarthritis, inflammatory arthritis, or rheumatoid arthritis were not included in this study. All patients who underwent medial UKA with the conventional or robot-assisted surgical procedure underwent clinical and radiologic assessment.

### Surgical procedures

All UKA procedures were performed by a single orthopedic surgeon using a standard medial parapatellar approach with tourniquet inflation. All UKA procedures were performed with the goal of possibly creating a 0-degree mechanical axis (mFTA).

Conventional UKA was performed using a medial Zimmer Unicompartmental High FlexKnee System (Zimmer Inc., Warsaw, USA). A medial capsulotomy was performed, followed by minimal medial soft tissue release. Osteophytes were removed and the distal femur and proximal tibia were visualized. The distal femoral cut was set to make a 4° valgus to the anatomical femoral axis using an intramedullary guide. The tibial cut was performed using an extramedullary guide aiming to be perpendicular to the tibial anatomical axis in the coronal plane. Since a posterior stabilized implant was used, the posterior slope of the proximal tibia was set to make a 5° posterior slope in the sagittal plane. Next, the femoral component, tibial component, and fixed bearing polyethylene liner of the medial Zimmer Unicompartmental High FlexKnee System were inserted.

In patients who received robot-assisted UKA, preoperative CT scans were performed to facilitate preoperative surgical planning. CT scan files of these patients were taken based on the Mako PKA CT scanning protocol for knee arthroplasty, which includes images of three regions (hip, knee, and ankle). These data (DICOM file format) were imported into the MAKOplasty^®^ Specialist laptop and segmented to build patient-specific 3-D bone models. The MAKOplasty^®^ Specialist (MPS) instrument selected CT landmarks that would be required for axes, bone registration, flexion/extension angles, varus/valgus angles, internal/external rotation angles, and implant alignment planning. The intraoperative registration process included fixation of dynamic referencing femur and tibia arrays and installation of checkpoints in the medial side of the medial femoral condyle and the anterior part of the medial tibial condyle. Next, dynamic soft tissue balancing was achieved by recording the flexion and extension gaps through the knee’s range of motion (0°, 30°, 60°, 90°, 120°). After proper component alignment was achieved using this preoperative plan, intraoperative registration, balancing, and bone preparation were performed using the RIO haptic guided robotic-arm. By creating virtual walls, a high-speed water-cooled burr was used to accurately resect bone within the predefined boundary. A tibial cut was performed, aiming to be perpendicular to the tibial anatomical axis in the coronal plane. Since Asians have a higher degree of posterior tibial slope compared to Westerners [29–31], the posterior slope of the proximal tibia was set to make a 7° posterior slope in the sagittal plane to conserve the native soft tissue balance of the knee. Next, the femoral component, tibial component, and fixed-bearing polyethylene liner of the RESTORIS MCK implant (Stryker Corp., Mahwah, NJ, USA) were inserted. In all cases, Polyethylene (PE) liner thickness was also measured.

### Clinical outcome evaluation

Clinical information was collected preoperatively and postoperatively from all patients using pre-designed datasheets in the outpatient clinic. This information was entered in our database by an independent investigator. The following preoperative clinical statuses and postoperative outcomes were evaluated: motion arc of the knee (flexion contracture, active flexion), American Knee Society (AKS) score [32], Western Ontario McMaster University Osteoarthritis Index scale score (WOMAC) [33], and Patellofemoral Feller (PF) score [34]. The motion arc of the knee was represented by maximum active flexion and range of motion (ROM), which was calculated by subtracting the degree of flexion contracture from the degree of maximum flexion. An independent investigator used a goniometer to measure flexion contracture and maximum flexion to the nearest 5° with the patient in the supine position. Preoperative clinical scores were assessed on the day before surgery by an independent investigator. Postoperative clinical scores were assessed at minimal 24 months after surgery, and these postoperative outcomes were compared.

### Radiologic outcome evaluation

Preoperative (day before surgery) and postoperative (i.e. follow-up visits of at least 24 months after surgery) radiologic examinations were performed by an independent investigator. The process consisted of standard anteroposterior and lateral radiographs of the knee, as well as anteroposterior and lateral radiographs of the entire lower extremity during weight bearing. Mechanical alignment was measured by calculating the mechanical femorotibial angle (mFTA) and performing the Kennedy protocol [35]. The mFTA is the angle subtended by a line extending from the center of the femoral head to the center of the knee joint to the center of the ankle mortise. An mFTA outlier was defined as an angle 3° outside of the optimum angle. To determine the Kennedy zone, a straight line was drawn from the center of the femoral head to the center of the ankle mortise. The number of patients with a mechanical axis lying in Kennedy’s central zone (zone C) or zone 2 was also calculated. The coronal femur and tibia alignments were determined using anteroposterior radiographs of the lower extremity long bone during weight bearing. The femoral component coronal alignment was measured as the angle between the femoral mechanical axis and the medial to lateral axis of the condylar implant. The tibial component coronal alignment was measured as the angle between the tibial mechanical axis and the medial to lateral axis of the tibial implant. The posterior slope of the tibial component was measured from lateral radiographs of the lower extremity long bone during weight bearing. The posterior slope of the tibial component was measured between the sagittal mechanical axis of the tibia and the horizontal axis of the tibial component. To reduce radiologic measurement error, two measurements were taken two weeks apart. The reliability of radiologic measurements was assessed by the intraclass correlation coefficient (ICC), and all radiologic measurements showed excellent agreement (above 0.80).

### Statistical analysis

Statistical analyses were performed using SPSS for Windows (version 20.0, SPSS, Inc, Chicago, IL); p values <0.05 were considered significant. The independent-sample t test was used to compare continuous variables. Fisher’s exact test and the Chi-square test were used to compare categorical data. A multivariate logistic regression test was performed to analyze influencing factors for the radiologic outlier (postoperative mFTA, mechanical axis in the central Kennedy zone, femoral component coronal outlier, tibial component coronal outlier).

## Results

The mean follow-up period of all patients was 27.8 months (range: 24–35 months). There was no significant difference in age or BMI between the robot-assisted UKA group and the conventional UKA group. All subjects had a diagnosis of degenerative osteoarthritis or osteonecrosis (Table 1).

**Table 1:** Demographic data.

	Robot-assisted UKA (n = 55)	Conventional UKA (n = 57)	*p*
Age (years)	64.8 (57–70)	68.4 (58–72)	0.518
Sex (Male/Female)	M: F = 11: 44	M: F = 7: 50	0.440
BMI (kg/m^2^)	25.5 ± 2.5 (20.59–30.33)	25.9 ± 3.7 (22.1–31.3)	0.545
Diagnosis (n)	Osteoarthritis (47) Osteonecrosis (8)	Osteoarthritis (44) Osteonecrosis (13)	

Table 2 summarizes the preoperative clinical and radiological data in the two groups. Comparison of the preoperative clinical outcomes revealed no significant differences between the two groups regarding flexion contracture, active flexion, AKS score, PF score, or WOMAC score.

**Table 2:** Preoperative clinical and radiologic data.

	Robot-assisted UKA (n = 55)	Conventional UKA (n = 57)	*p*
Flexion contracture	4.9° ± 4.5° (0°-20°)	4.1° ± 4.4° (0°-15°)	0.832
Active flexion	133.5° ± 10.5° (110°-150°)	134.8° ± 11.4° (120°-150°)	0.882
AKS knee score	56.6 ± 22.5 (40–75)	52.2 ±18.8 (42–70)	0.059
AKS function score	60.6 ± 13.0 (35–75)	57.3 ±11.5 (40–70)	0.142
PF score	22.6 ± 5.8 (0–30)	23.8 ± 4.0 (17–30)	0.115
WOMAC (total)	47.2 ± 19.8 (18–96)	50.2 ± 11.0 (21–70)	0.100
Pain	8.9 ± 4.1 (0–20)	9.6 ± 3.5 (4–16)	0.312
Stiffness	3.9 ± 1.9 (0–8)	3.8 ± 1.47 (1–6)	0.833
Functional	31.7 ± 14.1 (3–68)	36.8 ± 8.8 (16–52)	0.063
mFTA (varus)	5.7° ± 3.8° (-2°-10°)	4.5° ± 2.5° (1°-9°)	0.061

Data are presented as means ± standard deviations.

Abbreviations: AKS, American Knee Society; WOMAC, Western Ontario and McMaster Universities; PF, patellofemoral; mFTA, mechanical femorotibial axis.

The postoperative mean mechanical femorotibial angle was 1.2° ± 3.1° (range, -6° to 7°) of varus for the robot-assisted UKA group and 2.1° ± 4.8° (range, -5° to 6°) of varus for the conventional UKA group (Table 3); these values were not significantly different. However, the conventional UKA group (24/57) had more outliers (defined as an angle 3° away from the optimum angle) than the robot-assisted UKA group (12/55) (*p* = 0.022) (Table 3).

**Table 3:** Postoperative radiologic outcomes.

	Robot-assisted UKA (n = 55)	Conventional UKA (n = 57)	*p*
Mechanical alignment			
mFTA (varus)	1.2° ± 3.1° (-6°-7°)	2.1° ± 4.8°(-5°-6°)	0.105
Outliers*	12 (21.8%)	24 (42.1%)	**0.022**
MA in the central zone or Kennedy zone 2	49 (89.1%)	47 (82.4%)	0.316
MA in the central Kennedy zone	37 (67.3%)	21 (36.8%)	**<0.001**
Coronal Alignment			
Femoral component	90.8° ± 1.9° (85–98)	91.2°± 3.0° (84–99)	0.537
Outliers*	6 (10.9%)	15 (26.3%)	**0.037**
Tibial component	89.6°± 2.9° (87–93)	87.7°± 2.5° (85–94)	**0.045**
Outliers*	5 (9.1%)	18 (31.6%)	**0.003**
Posterior slope of the tibial component	7.8°± 1.8° (4°-9°)	4.5°± 2.9° (1°-8°)	**<0.001**

Abbreviations: mFTA, mechanical femorotibial axis; MA, mechanical axis.

*At least 3° outside of the optimum angle (optimum, 90°).

The Kennedy zone distributions of mechanical axis restoration after UKA were within the central zone or zone 2 in 89.1% of the patients in the robotic group and 82.4% of the patients in the conventional group; these rates were not significantly different (*p* = 0.121). Significantly more patients in the robotic group had a Kennedy zone within the central zone compared to the conventional group (67.3% *versus* 36.8%, *p*<0.001) (Table 3). Regarding component position, there was no significant difference in the coronal alignments of the femoral component between the two groups. However, the number of femoral component coronal alignment outliers was significantly different between the robotic group and conventional group (6/55 versus 15/57, p = 0.037) (Table 3). There was also a significant difference in the coronal alignments and posterior slopes of the tibial component (Table 3). In addition, there was a significant difference in the number of tibial component coronal alignment outliers (5/55 versus 18/57, *p* = 0.045).

Postoperatively, there was no significant difference in any of the short-term clinical outcomes (flexion contracture, active flexion, AKS score, WOMAC score, and PF score) between the two groups. This also held true for patients whose final follow-up occurred at 2 years (Table 4). Polyethylene (PE) liner thickness was also significantly different between the two groups (8.4 ± 0.8 mm in the robot group and 8.8 ± 0.9 mm in the conventional group, *p* = 0.035) (Table 4).

**Table 4:** Postoperative clinical outcomes.

	Robot-assisted UKA (n = 55)	Conventional UKA (n = 57)	*p*
Final follow-up (minimum 2 years)			
Flexion contracture	3.9° ± 4.4° (0°-10°)	3.1° ± 2.7° (0°-10°)	0.470
Active flexion	138.3° ± 10.0° (120°-150°)	138.8° ± 6.9° (120°-150°)	0.848
AKS knee score	83.6 ± 18.6 (75–95)	87.8 ± 8.3 (72–90)	0.066
AKS function score	68.4 ± 16.0 (50–90)	70.8 ± 13.1 (55–90)	0.381
PF score	22.3 ± 4.9 (17–30)	21.9 ± 4.5 (20–30)	0.642
WOMAC (total)	29.9 ± 18.1 (0–48)	27.8 ± 16.5 (3–54)	0.533
Pain	5.6 ± 4.4 (0–12)	4.7 ± 4.0 (0–16)	0.229
Stiffness	2.7 ± 1.7 (0–4)	2.4 ± 1.4 (0–6)	0.362
Functional	22.1 ± 13.5 (0–32)	21.0 ± 12.0 (3–32)	0.663
PE thickness (mm)	8.4 ± 0.8 (8–10)	8.8 ± 0.9 (8–10)	**0.035**

Abbreviations: AKS, American Knee Society; WOMAC, Western Ontario and McMaster Universities; PF, patellofemoral; PE, polyethylene.

The changes in preoperative and postoperative clinical scores were not different between the two groups, except for the AKS knee score. The difference between preoperative and postoperative AKS knee scores was significantly higher in conventional group compared to robotic group (27.5 ± 12.8 versus 34.3 ± 11.3, p = 0.004) (Table 5).

**Table 5:** Comparison of the change between preoperative and postoperative clinical scores.

	Robot-assisted UKA (n = 55)	Conventional UKA (n = 57)	*p*
AKS knee score(Δ)	27.5 ± 12.8	34.3 ± 11.3	**0.004**
AKS function score(Δ)	7.8 ± 21.2	13.5 ± 16.9	0.121
PF score(Δ)	-0.5 ± 7	-2 ± 5.6	0.164
WOMAC (total)(Δ)	34.8 ± 16.1	34.4 ± 12.5	0.084
Pain(Δ)	1.2 ± 1.9	1.4 ± 1.9	0.569
Stiffness(Δ)	1.2 ± 1.9	1.4 ± 1.9	0.099
Functional(Δ)	10.9 ± 15.3	15.6 ± 14.7	0.874

Data are presented as means ± standard deviations.

Abbreviations: AKS, American Knee Society; WOMAC, Western Ontario and McMaster Universities; PF, patellofemoral

Except for operation type (robotic or conventional), no other factors (age, sex, BMI, preoperative mFTA, preoperative AKS knee score, preoperative AKS function score, preoperative WOMAC score, and preoperative PF score) affected postoperative radiologic outliers in univariate model. Moreover, operation type (conventional UKA compared to robot-assisted UKA) was the only independent risk factor that influenced radiologic outliers (outlier of mechanical femorotibial angle, outlier of mechanical axis in central Kennedy zone, outlier of femoral component coronal alignment, and outlier of tibial component coronal alignment) (odds ratio: 2.833, 3.471, 5.160, 8.347; p = 0.037, 0.07, 0.013, 0.002) in multivariate model including age, sex, BMI, preoperative mFTA, preoperative AKS knee score, preoperative AKS function score, preoperative WOMAC score, and preoperative PF score (Table 6).

**Table 6:** Comparison of robot-assisted UKA and conventional UKA as risk factors of radiologic outliers (outlier of mechanical femorotibial angle, outlier of mechanical axis in central Kennedy zone, outlier of femoral component coronal alignment, outlier of tibial component coronal alignment) in multivariate regression analysis including age, sex, BMI, preoperative mFTA, preoperative AKS knee score, AKS function score, WOMAC score, and PF score.

Outcome		Multivariate	
		OR (95% CI)	P
Outlier of mFTA	Robotic	Reference	
	Conventional	2.833 (1.063–7.553)	**0.037**
Outlier of MA in central Kennedy zone	Robotic	Reference	
	Conventional	3.471 (1.406–8.566)	**0.007**
Outlier of femoral component coronal alignment	Robotic	Reference	
	Conventional	5.160 (1.410–18.876)	**0.013**
Outlier of tibial component coronal alignment	Robotic	Reference	
	Conventional	8.347 (2.153–32.366)	**0.002**

Abbreviations: mFTA, mechanical femorotibial axis; MA, mechanical axis; AKS, American Knee Society; WOMAC, Western Ontario and McMaster Universities; PF, patellofemoral

## Discussion

The most important finding of this study is that robot-assisted UKA achieved fewer outliers of mechanical axis and fewer outliers of femoral and tibial component position compared to conventional UKA. Although mechanical femorotibial axes were not significantly different between the two groups, fewer outliers were observed in robot-assisted UKA group. In both groups, most of the mechanical femorotibial axes were observed to be in the central zone or in Kennedy zone 2. However, considerably more mechanical femorotibial axes were observed in the central zone in robot-assisted UKA group. In addition, robot-assisted UKA was the only factor that lowered the radiologic outlier compared to conventional UKA in multivariate models that included age, sex, BMI, preoperative mFTA, and preoperative clinical scores. This study also found that patients who underwent robot-assisted UKA had reduced postoperative pain and better functional recovery at follow-up of at least 2 years, even though there was no significant difference between the two surgical procedures. In fact, several studies have shown that robot-assisted UKA improves component positioning and reduces radiologic outliers in early radiologic outcomes [17,21]. The current study also found similar results. Moreover, this is the first study to compare the clinical and radiologic outcomes of robot-assisted UKA and conventional UKA in Asian patients.

Since UKA involves resurfacing of the tibia and femoral components, it is important to avoid excessive correction of mechanical alignment, unlike the principle of TKA [36,37]. Achieving accurate tibial and femoral component alignment is critical for attaining favorable postoperative long-term surgical outcomes. In our study, since the instruments used in the two methods have many differences, it was not possible to perform an accurate comparison of component positioning using x-rays. The distal femoral bone cuts made in the two methods were different, precluding a comparison of femoral component sagittal alignments. However, it was possible to compare the tibial and femoral component coronal alignments. The femoral component should be aligned along the coronal mechanical axis, and the tibial component should be cut at a 90 degrees angle to the coronal mechanical axis. The femoral and tibial component coronal alignments were not significantly different between the two groups. However, despite the comparable mechanical femorotibial axes, fewer outliers were observed in the robot-assisted group. The operation time of the robot-assisted procedure was lengthened by 5 to 15 minutes because of additional procedures such as reference array insertion and registration steps.

Robot-assisted UKA was first introduced in 2006. Since then, data about postoperative clinical outcomes of robot-assisted UKA in Western patients have accumulated. In this study, although there was no significant difference in clinical outcomes over short term follow-up, better clinical outcomes would be expected with longer follow-up considering conversion to TKA. This is because the bone cutting in robot-assisted UKA is planned more accurately before the operation. In our study, patients who underwent robot-assisted UKA needed a thinner PE liner. This finding could mean that more accurate planning was achieved and less bone cutting was needed in robot-assisted UKA. Thinner PE liner thickness does not necessarily mean that bone cutting was minimized during UKA operation, as the degree of soft tissue release varies from patient to patient. Nevertheless, due to the significant difference in PE liner thickness between the two groups, we could infer that the bone cutting using burr in robotic group was less than the bone cutting using saw in conventional group. Previous studies have reported on some advantages of using thinner PE with the least possible tibial bone loss in UKA [38,39]; in this regard, if conversion to TKA after UKA is considered, robot-assisted UKA would be advantageous. Moreover, most of the previous studies on robot-assisted UKA included only Western patients. For these reasons, studies with longer follow-up are needed, especially those including Asian patients.

There were several limitations to our study. First, the medial Zimmer Unicompartmental High Flex Knee System (Zimmer Inc., Warsaw, USA) implant was used with standard manual procedures in the conventional UKA group, whereas the MAKO implant was used in robot-assisted UKA. Since the MAKO implant is not designed for conventional UKA, direct comparison of the two methods was not possible. Although both instruments use a fixed bearing system (unlike previous studies [21]), the different implants used in the two methods may have affected the surgical outcomes of UKA. The MAKO implant and the medial Zimmer Unicompartmental High Flex Knee System have different femoral component shapes and were designed for dissimilar posterior tibial slopes; therefore, it would not be meaningful to compare the femoral and tibial component sagittal alignments as an assessment of postoperative radiologic outcomes. Also, since robot-assisted and conventional UKA groups in this study were not randomized, there may have been some selection bias. In general, robot-assisted UKA were considered for patients with better preoperative function; therefore, the better improvement of delta score in conventional UKA group of our study could have appeared due to this selection bias. Since the patients in robot-assisted UKA group of our study had slightly better preoperative function (AKS score, WOMAC score), this may have influenced the comparison of postoperative outcomes between the two groups. Also, sample size was not performed, as this study was a retrospective analysis of 112 consecutive patients who underwent two types of UKA. Second, the 2-year follow-up period was relatively short. Although early postoperative surgical outcomes are important, longer follow-up data are needed to accurately evaluate the clinical and radiologic outcomes of robot-assisted UKA. Differences may emerge between the two methods with long-term follow-up. Due to the short follow-up period, survival rates, complication rates, knee arthritis rates of other compartments, and conversion rates to TKA could not be evaluated. Therefore, these patients should be studied over a longer follow-up period. Finally, postoperative computed tomography was not performed. This imaging tool could provide more accurate information regarding radiologic outcomes. Since rotational alignment could not be evaluated with x-rays, computed tomography would be helpful. Additionally, there was a risk of bias because the observers were aware of the surgical procedure method. To reduce this risk, radiologic assessment was performed by another clinical fellow.

## Conclusion

In summary, robot-assisted UKA has many advantages over conventional UKA, such as its ability to achieve precise implant insertion and reduce radiologic outliers. Although the clinical outcomes of robot-assisted UKA over a short-term follow-up period were not significantly different compared to those of conventional UKA, there has not been a study revealing the clinical superiority of robotic-assisted UKA despite improved radiologic outcomes, as we have done so in the current study. Notably, this study demonstrates that the findings of several Western studies, which reported excellence of robot-assisted UKA, are also applicable to Asian patients. Therefore, longer follow-up period is needed to determine whether the improved radiologic accuracy of the components in robotic-assisted UKA will lead to better clinical outcomes and improved long-term survival.

## Supporting information

S1 Dataset Minimal data set. (SAV)
